# Medical Professionals’ Opinions of and Attitudes Toward Uterus Transplantation in Hungary

**DOI:** 10.3390/clinpract15110194

**Published:** 2025-10-25

**Authors:** Kata Szilvia Papp, Peter Szakaly, Szilard Kolumban, Kálmán András Kovács, Jozsef Bodis, Nelli Farkas, Gabor Fazekas, Balint Farkas

**Affiliations:** 1 Department of Obstetrics and Gynaecology, University of Pécs Medical School, 7624 Pécs, Hungary; papp.kata@pte.hu (K.S.P.); kolumban.szilard@pte.hu (S.K.); kovacs.kalman@pte.hu (K.A.K.); bodis.jozsef@pte.hu (J.B.); farkas.balint@pte.hu (B.F.); 2 Doctoral School of Health Sciences, Faculty of Health Sciences, University of Pécs, 7621 Pécs, Hungary; 3 Department of Surgery, University of Pécs Medical School, 7624 Pécs, Hungary; szakaly.peter@pte.hu; 4 National Laboratory on Human Reproduction, University of Pécs, 7624 Pécs, Hungary; 5 Institute of Bioanalysis, University of Pécs Medical School, 7624 Pécs, Hungary; nelli.farkas@aok.pte.hu; 6 Department of Vascular Surgery, University of Pécs Medical School, 7624 Pécs, Hungary

**Keywords:** uterus transplantation, professional opinion, survey, Hungary

## Abstract

**Background**: Uterus transplantation (UTx) is a proven treatment for individuals affected by absolute uterine factor infertility (AUFI) who desire biological motherhood. Despite the fact that over 130 procedures have been performed worldwide in the past decade, UTx remains relatively unfamiliar, even among healthcare professionals. This study aimed to identify knowledge gaps regarding and evaluate attitudes toward UTx among Hungarian obstetricians/gynecologists and transplantation providers, in anticipation of the first procedure to be performed in the country. **Methods:** A Microsoft Forms^®^ questionnaire was distributed electronically among Hungarian medical professionals via e-mail, including members of the Hungarian Society of Obstetrics and Gynaecology and the Hungarian Transplantation Society. Additionally, participants of the “Update 2024” OB/GYN conference (held 28–29 November 2024, in Visegrád, Hungary) were invited to complete the survey through a QR code displayed during the event. **Results:** A total of 290 medical professionals completed the survey (response rate: 27.6%, 290/1050). Most of the respondents specialized in obstetrics and gynecology (81.7%, *n* = 237), with the remainder representing transplantation fields (18.3%, *n* = 53). Over half (56.6%, *n* = 161) reported they would recommend UTx to patients with AUFI, and 64.1% (*n* = 186) agreed that UTx should be available as a treatment option. The medical risks associated with the procedure were deemed acceptable for both living donors (58.0%, *n* = 168) and recipients (54.8%, *n* = 159). **Conclusions:** This is the first study to explore perceptions of UTx among Hungarian medical professionals. The findings suggest there is a generally favorable professional attitude toward its future clinical implementation.

## 1. Introduction

Absolute uterine factor infertility (AUFI) is a rare condition in which the uterus is either anatomically absent or functionally impaired, resulting in infertility [1]. For women affected by AUFI who desire motherhood, the only available options are adoption, surrogacy, or uterus transplantation (UTx). In Hungary, adoption is often a prolonged and highly bureaucratic process, while surrogacy remains illegal, making UTx a particularly viable alternative [2]. UTx has evolved from an experimental procedure into a proven clinical concept; by 2025, more than 130 UTx procedures had been performed across nearly 20 centers in Europe, North America, Latin America, and Asia, resulting in over 70 live births [3]. UTx was incorporated into the national healthcare system in Germany in 2020 [4,5], and an international quality registry was established by the International Uterus Transplantation Society (ISUTx) in the same year [6]. Although the first successful live birth following UTx occurred a decade ago [7], the procedure has not yet been introduced in Hungary.

Only a limited number of studies—from the United States, the United Kingdom, and Canada—have investigated healthcare professionals’ perspectives on the acceptance and desirability of UTx [8,9,10,11,12]. These studies revealed divergent opinions: while a majority of respondents supported UTx as a treatment option for patients with AUFI, several concerns were raised regarding the ethical aspect of the procedure, including the risk of medical and surgical complications. Moreover, some respondents characterized UTx as a non-vital and non-quality-of-life-enhancing organ transplant [8,9,10,11,12]. As UTx has progressed from being an early-experimental-phase treatment to a well-developed surgical technique, awareness among healthcare providers has increased, and overall attitudes have become more favorable-though regional differences in perception persist [8,9,10,11,12].

Our multidisciplinary research team is committed to establishing UTx as an available treatment option for patients with AUFI in Hungary. In preparation for clinical implementation, we have conducted a series of preclinical studies involving both animal models and human cadavers [13,14]. The aim of the present study was to assess the perceptions and attitudes of obstetricians/gynecologists and professionals associated with transplantation within the Hungarian medical community. These two specialist groups were selected because of their likely involvement in future UTx procedures, and their points of view, risk assessments, and professional opinions may differ. Insights gained from this study will inform professional discourse and support the development of national strategies to introduce UTx as a treatment modality for AUFI in Hungary.

## 2. Materials and Methods

### 2.1. Survey Development

This cross-sectional questionnaire-based study was conducted by the Department of Obstetrics and Gynecology at the University of Pécs, Hungary. Since the survey has been completed anonymously by medical professionals and did not involve patient data, ethical approval from the University of Pécs Institutional Ethical Review Board was not applicable.

An online survey was developed using the Microsoft Forms^®^ platform (Microsoft Corporation, Redmond, WA, USA), based on a previously published questionnaire assessing healthcare professionals’ perceptions of uterus transplantation in the United States. Permission to adapt the original survey was obtained from the corresponding author [10].

The questionnaire was translated into Hungarian, the official language of the country, and culturally adapted. It included an introduction of the study conductor, a brief professional overview of UTx, six demographic questions, and eleven perception-related statements assessed using a 5-point Likert scale ranging from “strongly disagree” to “strongly agree” (Appendix A). Prior to widespread distribution, a pilot test was conducted to evaluate the content validity and clarity of the survey; however, no formal psychometric validation was performed.

### 2.2. Survey Distribution

The survey was distributed among Hungarian medical professionals via e-mail (members of the Hungarian Society of Obstetrics and Gynaecology, and the Hungarian Transplantation Society), and the attendees of a Hungarian OB/GYN conference (Update 2024, 29–30 November 2024) were invited to participate by displaying a QR code at the end of an oral presentation on UTx concept (by BF, KK, SR) allowing them to complete the survey on their personal mobile devices.

In total, 1050 individuals were contacted via e-mail, of whom 594 opened the message. Including both e-mail recipients and conference attendees, a total of 290 Hungarian medical professionals completed the survey (response rate 27.6%). No a priori sample size calculation was performed, given the exploratory design. At a 95% confidence level and assuming *p* = 0.5, the margin of error with finite population correction for *n* = 290 and *N* = 1050 is ±4.9%. The data collection interval took place from 15 October 2024 to 11 January 2025.

Inclusion criteria were: (i) licensed physicians practicing in Hungary at the time of the survey; (ii) age ≥ 18 years; and (iii) provision of informed consent. Exclusion criteria were: (i) medical students; (ii) duplicate submissions (screened and removed prior to analysis); and (iii) questionnaires with >50% missing items in the Likert section. Practice status was self-reported in the demographic section; all included respondents affirmed current practice at the time of the survey.

### 2.3. Statistical Analysis

Descriptive statistics were used to summarize and present the characteristics of the study sample and variables. All examined variables were categorical data, so frequencies and percentages were reported.

To assess associations between the categorical variables, the Chi-squared test was primarily used. Where the assumptions for the Chi-squared test were not met, Fisher’s exact test was applied for increased accuracy in evaluating statistical significance. A *p*-value < 0.05 was considered statistically significant.

All statistical analyses were conducted using R software (version 4.2.1, R Core Team (2023). R: A Language and Environment for Statistical Computing. R Foundation for Statistical Computing, Vienna, Austria. Available online: https://www.R-project.org/ (accessed on 9 April 2025)).

## 3. Results

### 3.1. Demographic Characteristics

The demographic characteristics of the study participants are summarized in Table 1. A total of 290 respondents completed the survey. Male participants comprised the majority (67.2%, *n* = 195), while female participants accounted for 32.8% (*n* = 95). All age groups were approximately equally represented (21%, 20%, 21%, 21%, and 18%, respectively).

In terms of professional qualification, the vast majority of respondents were medical specialists (90.0%, *n* = 261). Most participants specialized in obstetrics and gynecology (81.7%, *n* = 237). Among these, 81.4% (*n* = 193) identified as general obstetricians and gynecologists, 8.0% (*n* = 19) as infertility specialists, 6.7% (*n* = 16) as gynecologic oncologists, and 3.8% (*n* = 9) as adolescent gynecologists (subgroups not shown in table). Approximately one-fifth of all respondents (18.3%, *n* = 53) were transplant surgeons.

For analytical purposes, participants were grouped into broader categories based on their primary area of specialization: “OB/GYN,” “Transplantation,” and “Other.” Similarly, workplace settings were consolidated into six main categories. Nearly half of respondents reported working in a clinical center (42.4%, *n* = 123), while one-third were employed in city or county hospitals (32.8%, *n* = 95) (Table 1).

### 3.2. Candidates for UTx

**Figure 1:**
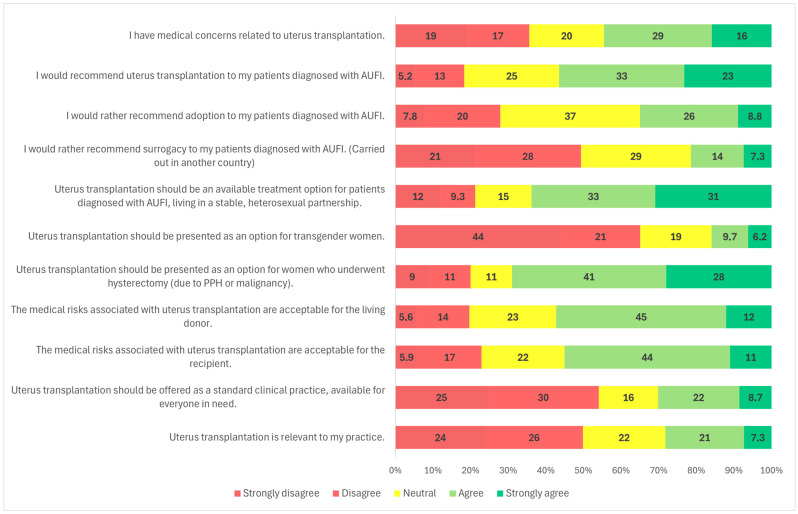
Overall survey results.

**Figure 2:**
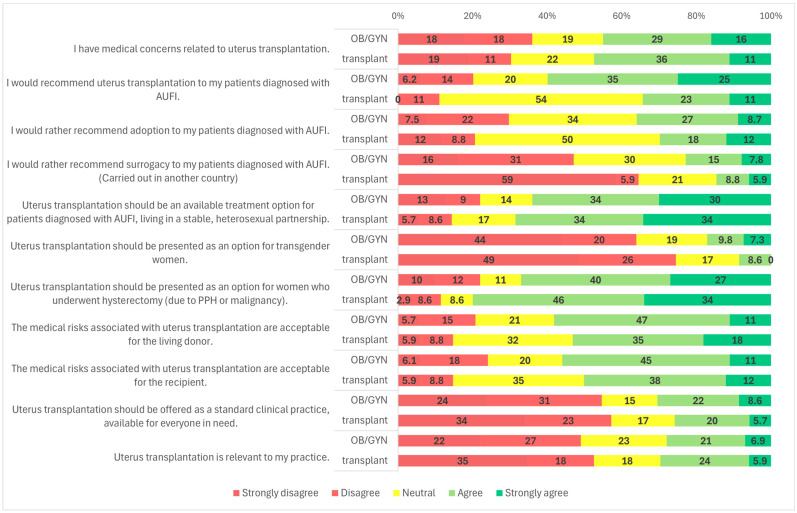
Survey results by OB/GYN or transplant specialty.

**Figure 3:**
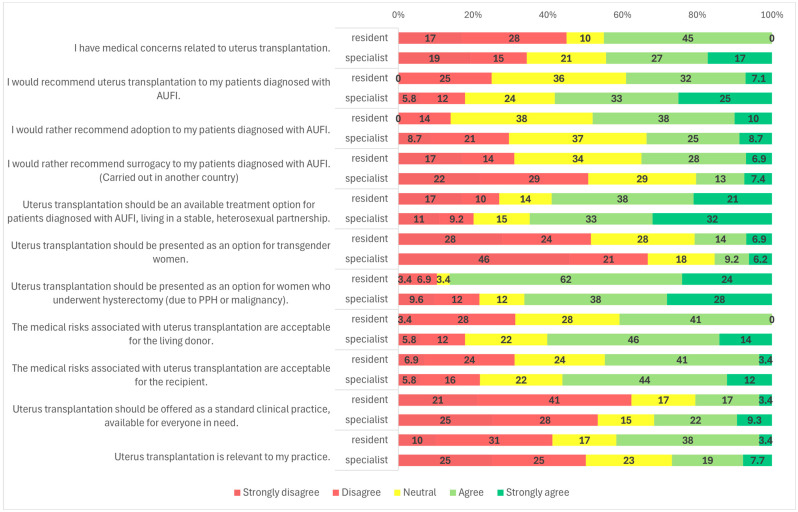
Survey results by medical qualification.

**Figure 4:**
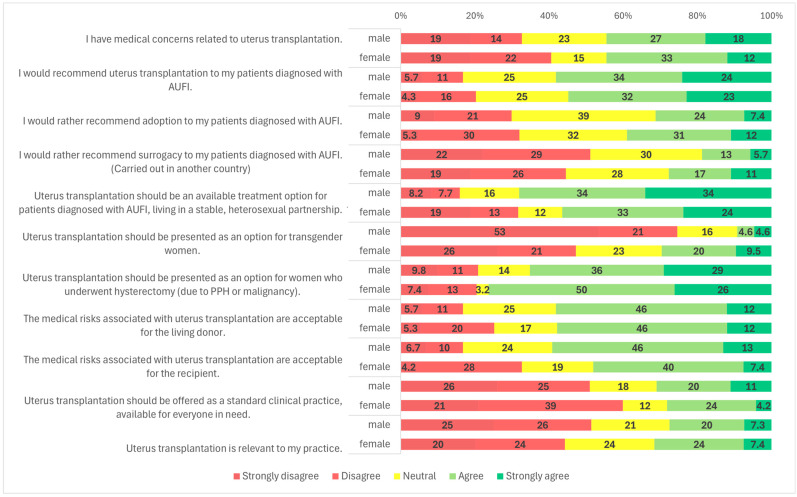
Survey results by gender groups.

**Figure 5:**
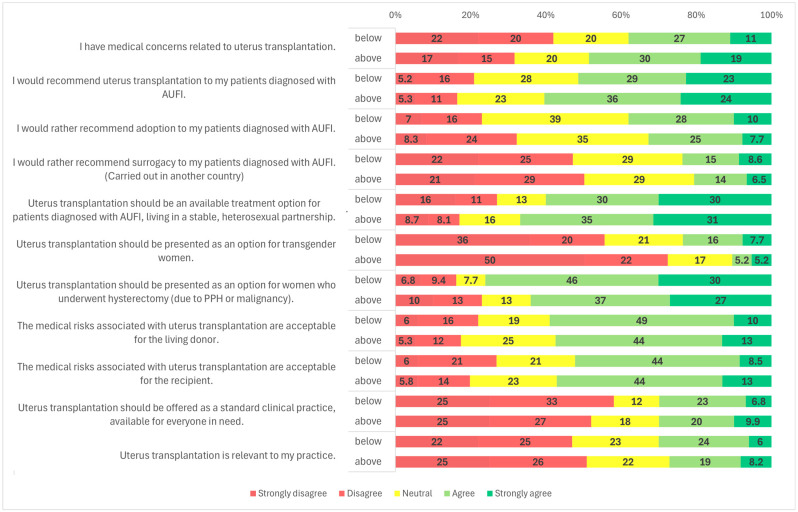
Survey results by age groups-below or above 45 years.

Survey responses indicated that 56.6% (*n* = 162/286) of participants agreed or strongly agreed that they would recommend UTx to their patients (Figure 1). When analyzed by medical specialty, a significantly higher proportion of OB/GYN specialists (59.9%, *n* = 145/242) supported recommending UTx compared to transplantation professionals (34.3%, *n* = 12/35) (*p* = 0.001) (Figure 2). Similarly, specialists were more likely to recommend UTx than residents (58.5%, *n* = 151/258 vs. 39.3%, *n* = 11/28; *p* = 0.036) (Figure 3). No statistically significant difference was observed between male and female respondents in this regard (*p* = 0.857) (Figure 4), and participants’ age had no significant impact on responses as well (Figure 5).

Participants were also asked about potential target populations for UTx. The most widely supported group was patients diagnosed with AUFI who are in stable, heterosexual relationships; 64.0% (*n* = 185/289) of respondents agreed with this indication (Figure 1). A statistically significant gender difference was observed: 68.0% (*n* = 131/194) of male respondents and 56.8% (*n* = 54/95) of female respondents supported this indication (*p* = 0.025) (Figure 4). No significant variation was found based on respondents’ age, medical specialty, or medical qualification (Figure 2, Figure 3 and Figure 5).

In contrast, support for offering UTx to transgender women was considerably lower. A majority of respondents (65.4%, *n* = 189/289) disagreed with offering UTx to this group, while a significant proportion, 19.7% (*n* = 57/289) remained neutral, and only 15.9% (*n* = 46/289) were supportive (Figure 1). A statistically significant gender difference was found (*p* < 0.001): 29.5% (*n* = 28/95) of female respondents expressed support, compared to only 9.3% (*n* = 18/194) of males. Additionally, 47.4% (*n* = 45/95) of women and 74.2% (*n* = 144/194) of men opposed UTx for transgender women (Figure 4).

Age also significantly influenced attitudes toward this target group (*p* = 0.011). Among respondents younger than 45 years, 23.9% (*n* = 28/117) supported UTx for transgender women, 20.5% (*n* = 24/117) remained neutral, and 55.6% (*n* = 65/117) opposed it. Among those older than 45 years, only 10.5% (*n* = 18/172) expressed support, 17.4% (*n* = 30/172) were neutral, and 72.1% (*n* = 124/172) disagreed with offering UTx to transgender women (Figure 5).

Another subgroup presented was patients with AUFI due to hysterectomy following malignancy or postpartum hemorrhage. This group received broad support, with 68.9% (*n* = 199/289) of respondents indicating they would recommend UTx for these patients (Figure 1). Gender differences were again statistically significant (*p* = 0.021): 76.8% (*n* = 73/95) of female respondents and 64.9% (*n* = 126/194) of male respondents supported this indication. Neutral responses were less common among women (3.2%, *n* = 3/95) than among men (14.4%, *n* = 28/194) (Figure 4). No significant differences were observed based on specialty, age, or medical qualification (Figure 2, Figure 3 and Figure 5).

### 3.3. Alternative Treatment Options for AUFI

Participants were asked whether they would rather recommend an alternative treatment option for patients with AUFI, such as adoption or surrogacy.

Adoption, a legal alternative in Hungary, was supported by 34.8% of respondents (*n* = 99/283), while 37.0% (*n* = 104/283) were neutral and 27.8% (*n* = 80/283) disagreed (Figure 1). There were no statistically significant differences in responses based on gender, specialty, or age (Figure 2, Figure 4 and Figure 5).

Regarding surrogacy, which is illegal in Hungary and many European Union countries, nearly half of participants (49.0%, *n* = 140/286) stated they would not recommend it over UTx. Approximately 29.4% (*n* = 84/286) were neutral, and 21.3% (*n* = 62/286) indicated they would support it despite its legal status (Figure 1). A statistically significant difference was observed based on medical specialty (*p* < 0.001): among OB/GYN specialists, 46.9% (*n* = 114/243) were opposed to surrogacy, compared to 64.7% (*n* = 22/34) of transplantation specialists. The proportion of neutral responses was relatively high, with 30.5% (*n* = 74/243) of OB/GYN providers and 20.6% (*n* = 7/34) of transplantation professionals selecting a neutral option. Only 22.6% (*n* = 55/243) of OB/GYN respondents and 14.7% (*n* = 5/34) of transplant specialists supported surrogacy as an alternative to UTx (Figure 2). No significant differences were observed based on gender, age, or medical qualification (Figure 3, Figure 4 and Figure 5).

### 3.4. Risk Assessment of the UTx Procedure

Participants were asked if they had medical concerns related to uterus transplantation. Nearly half (44.3%, *n* = 128/289) agreed with the statement (Figure 1). A statistically significant difference was found based on medical qualification (*p* = 0.009): 45.0% (*n* = 13/29) of residents disagreed with the statement, compared to only 34.6% (*n* = 90/260) of specialists (Figure 3). No significant differences were observed based on gender, age, or specialty (Figure 2, Figure 4 and Figure 5).

Respondents were also asked whether they found the medical risks associated with living donors in UTx acceptable. Overall, 58.0% (167/288) agreed (Figure 1). The proportion was higher among specialists than residents (59.8% vs. 41.4%). Using a one-sided Fisher’s exact test, this difference was statistically significant (*p* = 0.044), consistent with our a priori expectation that specialists would be more supportive. No significant differences were observed by primary specialty, gender, or age (Figure 2, Figure 4 and Figure 5; Supplementary Data S1). Regarding acceptance of medical risks to the recipient, 55.2% (*n* = 159/288) of respondents agreed that these were acceptable (Figure 1). A statistically significant difference was found based on gender (*p* = 0.001): 59.1% (*n* = 114/193) of male respondents agreed, compared to 47.4% (*n* = 45/95) of female respondents. Additionally, 33.7% (*n* = 32/95) of women and 17.1% (*n* = 33/193) of men disagreed with the statement (Figure 4). No significant differences were observed based on age, specialty, or medical qualification (Figure 2, Figure 3 and Figure 5).

### 3.5. UTx as Standard Clinical Practice

Participants were asked whether uterus transplantation should be offered as a standard clinical treatment, available to all eligible patients. The majority (54.2%, *n* = 156/288) disagreed with this statement, while only 30.2% (*n* = 87/288) agreed (Figure 1).

A significant gender-based difference was observed (*p* = 0.037): 60.0% (*n* = 57/95) of female respondents disagreed, compared to 51.3% (*n* = 99/193) of male respondents (Figure 4). No statistically significant differences were found based on age, specialty, or medical qualification (Figure 2, Figure 3 and Figure 5).

### 3.6. Relevance to Practice

The final survey item assessed whether participants considered UTx relevant to their clinical practice. Nearly half of the respondents (49.3%, *n* = 142/288) disagreed with the statement, while 22.2% (*n* = 64/288) provided a neutral response, and 28.5% (*n* = 82/288) agreed (Figure 1). No statistically significant differences were observed based on gender, age, medical qualification, or medical specialty (Figure 2, Figure 3, Figure 4 and Figure 5).

## 4. Discussion

To date, only a limited number of studies have explored medical professionals’ perspectives on UTx [8,9,10,11,12]. These studies have examined perceptions in countries such as the United States, Canada, and the United Kingdom. In a 2018 U.S. study, 56% of reproductive endocrinologists and gynecologic surgeons supported UTx, with 45% considering it ethical [8]. A more recent U.S. survey in 2024 reported increased support, with 76% agreeing that UTx should be an available option for patients with congenital AUFI [10]. In Canada, 53.5% of OB/GYNs supported the concept of UTx donation and reception, though only 42.4% considered it a viable treatment option [12]. Across all studies, common concerns centered around medical and ethical risks to donors, recipients, and offspring. The Canadian study, for example, revealed more reluctance to accept donor-related risks compared to U.S. providers [12]. Notably, 68% of U.S. respondents in the 2024 survey supported offering UTx to transgender women [10].

Our findings align with those found in the currently available literature. In our Hungarian cohort, 56% of respondents were willing to recommend UTx to patients, and 64% agreed that the procedure should be an available option for individuals diagnosed with AUFI, despite it not yet being available in Hungary.

Although UTx is not a life-saving procedure, it offers significant quality-of-life benefits by enabling gestation and childbirth. Ethical concerns primarily involve surgical risks to healthy donors and recipients, both of whom undergo extensive operations-donor procedures typically lasting around 10 h, and recipient surgeries ranging from 2 to 6 h [15]. Donor complications include urinary tract injuries and postoperative issues requiring surgical intervention. A 2023 report documented a 17% rate of Clavien–Dindo grade III–IV complications among living donors (9/54), including hydronephrosis, ureteric fistula, and hypotonic bladder [15].

Recipients face a different risk profile, often related to vascular complications such as graft thrombosis, ischemia, necrosis, or intrauterine infection, which may necessitate hysterectomy prior to childbirth [15]. Immunosuppression after transplantation is a highly debated risk as well.

The use of deceased donors in UTx offers technical and ethical advantages [16,17,18]. Dissection of uterine arteries, veins, ureters, and paracervical tissue is avoided, making deceased donor surgeries shorter. Additionally, longer vascular pedicles and vaginal cuffs can be harvested, which may improve graft quality. Despite these advantages, debate persists over the preferred donor model. Current data suggest better outcomes with living donors [15], but ethical considerations-namely avoiding harm to healthy individuals-make the deceased donor model an attractive alternative. These perspectives must be carefully weighed before initiating a national UTx program in Hungary.

To date, UTx has been offered exclusively to cisgender women. Our study found strong disapproval of extending UTx to transgender women: 65% of respondents stated they would not support this indication. This contrasts sharply with findings from the most recent U.S. survey, where 68% of respondents supported offering UTx to transgender patients [10]. One explanation for this discrepancy may be Hungary’s limited exposure to UTx as a treatment option, given its unavailability. Another contributing factor may be Hungary’s sociopolitical environment, which is currently less supportive of the LGBTQ+ community.

This study has several limitations. In particular, although the questionnaire was adapted from a previously published survey and pilot-tested for clarity, it was not formally psychometrically validated. Although gender was recorded, it was captured in binary form (male/female) without non-binary options, and sex assigned at birth was not assessed. A key limitation of this survey is the risk of selection bias. Only short of 300 of more than 1000 contacted individuals responded, and respondents were disproportionately OB/GYN-affiliated. Also, the overall response rate was relatively low (27.6%), and the sample may not be fully representative of all Hungarian medical professionals who could be involved in future UTx programs. Additionally, only 28% of respondents indicated that UTx was relevant to their current practice. Despite these limitations, this study has notable strengths. It is the first published survey investigating Hungarian medical professionals’ attitudes toward UTx. The insights gained-particularly regarding risk assessment and ethical considerations-can guide preparatory efforts before clinical implementation. Furthermore, the age distribution of respondents was balanced, providing perspectives across generations and levels of experience.

## 5. Conclusions

UTx represents an innovative and promising treatment for individuals with AUFI who desire biological motherhood. As surrogacy remains illegal in Hungary and much of Europe, UTx may be the only path to gestation for many patients. This survey demonstrates general support among Hungarian medical professionals for the introduction of UTx, with the majority willing to recommend it and accepting its associated medical risks for both donors and recipients.

However, the relatively high proportion of neutral responses indicates a degree of uncertainty or knowledge gaps that must be addressed. Continued professional education and ethical discourse will be essential for the responsible integration of UTx into clinical practice in Hungary.

## Figures and Tables

**Table 1:** Demographic characteristics of study participants.

		Number of Participants (*n*)	Percentage (%)
**Gender**	Male	195	67.2
	Female	95	32.8
**Age**	25–35	60	20.6
	36–45	58	20
	46–55	61	21
	55–65	60	20.6
	>66	51	17.6
**Medical qualification**	Specialist	261	90
	Resident	29	10
**Years of experience**	0–5	30	10.3
	6–10	38	13.1
	11–15	28	9.6
	16–20	25	8.6
	21–25	32	11
	>26	137	47.2
**Medical specialty**	Anesthesiology	1	0.3
	GP	1	0.3
	Internal Medicine	2	0.7
	OB/GYN	245	84
	Surgery	1	0.3
	Transplantation	36	12
	Vascular surgery	4	1.4
**Professional setting**	Clinical centre	123	42
	City/County hospital	95	33
	Polyclinic	20	6.9
	Private care	46	16
	Fertility center	4	1.4
	Blood bank	2	0.7

## Data Availability

The original data presented in the study are openly available in FigShare at https://doi.org/10.6084/m9.figshare.29890742.v1 (accessed on 12 August 2025).

## Supplementary Materials

The following supporting information can be downloaded at: https://www.mdpi.com/article/10.3390/clinpract15110194/s1, Supplementary Data S1: Dataset containing anonymized survey responses used for statistical analysis.

## Abbreviations

The following abbreviations are used in this manuscript:

AUFI	absolute uterine factor infertility
MRKH	Mayer-Rokitansky-Küster-Hauser syndrome
UTx	uterus transplant
LD	live donor
DD	deceased donor

## Appendix A

### Appendix A.1. Background Information

Uterus transplantation (UTx) is a proven treatment for women impacted by absolute uterine-factor infertility (AUFI). The cause of AUFI can be congenital (MRKH sy.) or acquired-such as hysterectomy due to postpartum hemorrhage or malignancy, or Asherman-sy. The temporary graft (removed after childbirth) allows these women who could not be treated previously to carry a pregnancy and bear their own child. The alternative option for these patients to start a family is gestational surrogacy, which is illegal in Hungary (and most of Europe) or adoption which is often a slow and bureaucratic process.

The first successful uterus transplant in the world took place in Sweden in 2013, and a healthy baby boy was born in 2014. Since then an approximate of 100 procedures has been performed worldwide. UTx is not yet available in Hungary.

This procedure begins with the surgical removal of the donor uterus, either as an open procedure or as a complete/partial robotic-assisted procedure. The graft can be donated from either a living or deceased donor. Once the organ is transplanted into the recipient, the recipient begins immunosuppression to avoid graft rejection. Pregnancy is achieved through assisted reproductive technology.

All deliveries are currently being performed via cesarean section due to concerns of vaginal anastomosis and damage to structures during labor. Presently, a transplanted uterus is only utilized for a maximum of two to three pregnancies. Patients will then undergo a graft hysterectomy in order to discontinue immunosuppressive medications.

### Appendix A.2. Survey

*Demographics*

1. **What is your specialty? (multiple choice)**
  ☐ Obstetrician/gynecologist
  ☐ Assisted reproduction specialist
  ☐ Gynecological oncologist
  ☐ Pediatric and adolescent gynecologist
  ☐ Transplant surgeon
  ☐ Fetal ultrasound specialist
  ☐ Other (please specify)
2. **What is your current professional role?**
  ☐ Resident
  ☐ Specialist
3. **Gender**
  ☐ Male
  ☐ Female
4. **How old are you?**
  ☐ 25–35 years old
  ☐ 36–45 years old
  ☐ 46–55 years old
  ☐ 56–55 years old
  ☐ 66+ years old
5. **How long have you been practicing medicine?**
  ☐ 0–5
  ☐ 6–10
  ☐ 11–15
  ☐ 16–20
  ☐ 21–25
  ☐ 26+ years
6. **What best describes the medical setting you practice in? (multiple choice)**
  ☐ University clinic
  ☐ County hospital or teaching hospital
  ☐ City hospital
  ☐ Polyclinic
  ☐ Private clinic
  ☐ Private hospital
  ☐ Private practice
  ☐ Fertility center
  ☐ Other (please specify)

**Please respond to the following statements: (Strongly disagree, Disagree, Neutral, Agree, Strongly agree)**

1. I have medical concerns related to uterus transplantation.
2. I would recommend uterus transplantation to my patients diagnosed with AUFI.
3. I would rather recommend adoption to my patients diagnosed with AUFI.
4. I would rather recommend surrogacy to my patients diagnosed with AUFI. (Carried out in another country)
5. Uterus transplantation should be an available treatment option for patients diagnosed with AUFI, living in a stable, heterosexual partnership.
6. Uterus transplantation should be presented as an option for transgender women.
7. Uterus transplantation should be presented as an option for women who underwent hysterectomy (due to PPH or malignancy).
8. The medical risks associated with uterus transplantation are acceptable for the living donor.
9. The medical risks associated with uterus transplantation are acceptable for the recipient.
10. Uterus transplantation should be offered as a standard clinical practice, available for everyone in need.
11. Uterus transplantation is relevant to my practice.
